# Comparative accuracy of automated carbohydrate estimation using NutriDish and ChatGPT-5 against dietitian-derived reference values

**DOI:** 10.3389/fnut.2026.1927840

**Published:** 2026-09-29

**Authors:** Xia Lian, Weixiang Lian, Hwee Pin Phua, Alvin Wai Kit Tan, Huiling Liew, Eunice Yu Wen Goh, Christina Yang Hoon Goh, Melissa Hui Li Ho, Puay Shi Ng, Isaac Jun Song Tan, Helen Lim, Sanchalika Acharyya, Jocelyn Han Shi Chew, Wenru Wang, Rinkoo Dalan

**Affiliations:** 1 Department of Nursing, Tan Tock Seng Hospital, Singapore, Singapore; 2 Department of Endocrinology, Tan Tock Seng Hospital, Singapore, Singapore; 3 Alice Lee Center for Nursing Studies, Yong Loo Lin School of Medicine, National University of Singapore, Singapore, Singapore; 4 Department of Epidemiology & Preventive Medicine, Tan Tock Seng Hospital, Singapore, Singapore; 5 Department of Nutrition & Dietetics, Tan Tock Seng Hospital, Singapore, Singapore; 6 Clinical Research & Innovation Office, Tan Tock Seng Hospital, Singapore, Singapore; 7 Lee Kong Chian School of Medicine, Nanyang Technological University, Singapore, Singapore; 8 KLARIS Precision Health Research Clinic, Department of Endocrinology, Tan Tock Seng Hospital, Singapore, Singapore

**Keywords:** artificial intelligence, decision support system, dietary carbohydrate, food analysis, mobile application

## Abstract

**Background:**

Accurate carbohydrate estimation is critical for insulin dosing in individuals with diabetes on multiple daily injections. Conventional methods such as visual estimation and food exchange systems are inconsistent, particularly in multicultural Asian settings where portion sizes vary and nutritional labeling is limited. NutriDish is an AI-powered system designed to automates carbohydrate estimation from food images.

**Objective:**

This study compared the accuracy of carbohydrate estimates generated by NutriDish and ChatGPT-5 against reference values established by a dietitian using weighed food portions.

**Methods:**

Between July and August 2025, food items were collected from hawker centers, food courts, restaurants, and homes, supermarkets, and convenience stores across Singapore to capture the region's diverse culinary landscape. Items were classified as simple foods or composite meals. NutriDish and ChatGPT-5 independently estimated carbohydrate content, while a dietitian established reference values using weighing portions and a validated nutrient database. Accuracy was assessed using Mean Absolute Error (MAE), Mean Absolute Percentage Error (MAPE), proportion of estimates within ± 10 g of the reference value, and Bland-Altman analysis.

**Results:**

Across 150 food items, NutriDish achieved significantly lower MAE [5.9 ± 5.3 g (median: 4.1 g, IQR: 1.0–9.0) vs. 8.7 ± 7.4 g (median: 6.0 g, IQR: 3.0–13.4); *p* < 0.001] and MAPE [22.1 ± 35.7% (median: 13.0%, IQR: 4.9–25.0) vs. 32.9 ± 50.8% (median: 22.1%, IQR: 9.1–37.1); *p* < 0.001] than ChatGPT-5. NutriDish estimates fell within ± 10 g of the reference values in 80.7% of cases vs. 69.3% for ChatGPT-5 (*p* = 0.016). Simple food items were estimated more accurately than composite meals, with MAEs of 4.1 ± 4.3 g and 8.6 ± 5.6 g, respectively (*p* < 0.001). Regarding reference object types, nutrition labels yielded the highest accuracy (MAE: 2.7 ± 4.2 g; MAPE: 9.0% ± 14.5%), whereas coins were associated with the poorest performance (MAE: 9.3 ± 7.3 g; MAPE: 48.1% ± 74.6%).

**Conclusion:**

NutriDish demonstrated good agreement with dietitian-derived carbohydrate estimates, highlighting its potential to aid carbohydrate counting in individuals treated with insulin. Future studies should evaluate real-world clinical usability, integration into diabetes self-management ecosystems and impact on long-term glycemic control.

## 1 Introduction

Diabetes mellitus represents a significant and growing public health burden in Singapore, affecting approximately 10% of adults aged 18–69 years and nearly one-third of those aged over 60 years, with type 2 diabetes accounting for approximately 90% of all cases (1, 2). The rising prevalence is driven by a confluence of population aging, ethnic predisposition, particularly among Malay and Indian communities, and contemporary lifestyle factors. For individuals with type 1 diabetes managed on multiple daily insulin injections (MDII), accurate carbohydrate estimation is a cornerstone of effective self-management, as prandial insulin dosing is directly calibrated to carbohydrate intake (3, 4). Estimation errors within ± 10 g are generally considered to have minimal impact on glycemic outcomes; however, inaccuracies of approximately ± 20 g can significantly affect postprandial glycaemia and increase the risk of both hypoglycemia and hyperglycemia (5, 6). Beyond the clinical consequences, carbohydrate counting imposes a considerable psychological burden, with overestimation risking postprandial hypoglycemia and underestimation potentially resulting in sustained hyperglycemia (7, 8).

Singapore's distinctive food culture presents particular challenges for carbohydrate estimation in daily practice. Meals are predominantly consumed outside the home across hawker centers, food courts, and restaurants, where high-carbohydrate rice- and noodle-based dishes predominate, sweetened beverages are widely consumed, portion sizes vary significantly, and nutritional labeling is largely absent. The diversity of multi-ethnic cuisines, spanning Chinese, Malay, Indian, and Peranakan culinary traditions, further complicates accurate carbohydrate quantification. Despite structured diabetes education, many individuals continue to struggle with carbohydrate estimation (9–13), with conventional approaches such as manual weighing, calculation, and application-based tools remaining time-consuming and prone to inaccuracies arising from portion-size misjudgment, incomplete food databases, and the inherent variability of mixed dishes. This challenge is particularly pronounced in Southeast Asian populations, whose traditional diets are characteristically carbohydrate-dominant, with staple foods such as white rice, noodles, and rice-based dishes contributing substantially to daily intake. In Singapore, the National Nutrition Survey reported that carbohydrates accounted for 47% of total energy intake among adults aged 18–69 years, equivalent to approximately 279 g per day, consistent with the upper range of the recommended 45–50% carbohydrate-to-energy ratio, underscoring the importance of accurate carbohydrate estimation in this population, particularly for individuals managing diabetes (14).

Over the past decade, growing interest in artificial intelligence (AI)-assisted dietary assessment has driven the development of smartphone applications and automated carbohydrate estimation systems aimed at reducing the cognitive burden of diabetes self-management (15–19). Tools such as GoCARB (University of Bern, Bern, Switzerland) and SNAQ app (SNAQ GmbH, Zurich, Switzerland) have been evaluated with promising results (15, 16); however, their validation has been conducted predominantly in Western and Nordic food environments, with limited representation of Asian cuisines and the complex mixed dishes characteristic of communal dining settings in Singapore and comparable urban Asian contexts. This gap is clinically significant, as Asian meals frequently comprise multiple food components, varied preparation methods, and shared meal formats that differ substantially from the dietary patterns on which most existing tools have been developed and validated.

Recent advances in multimodal large language models (LLMs) have further expanded the potential for image-based carbohydrate estimation. Emerging studies have demonstrated promising performance using models such as ChatGPT-4o (OpenAI, San Francisco, CA, USA) for food image analysis (20, 21), with capabilities encompassing food item identification, portion size estimation, and conversion of estimated food weights into nutritional components, as demonstrated by Lo et al. (22). Accuracy has been shown to improve when additional contextual meal information is provided alongside images, highlighting the limitations of image-only approaches for mixed meals and real-world dining situations. Tecce et al. (23) further demonstrated that generative AI (GenAI) models such as ChatGPT and Gemini Advanced (Google LLC, Mountain View, CA, USA) could standardize and simplify carbohydrate counting by incorporating factors that influence postprandial glucose levels, with ChatGPT outperforming Gemini particularly under moderate data conditions and with prepackaged foods. Nevertheless, existing studies have also emphasized the need for cautious clinical application, as substantial carbohydrate misestimation may lead to insulin-carbohydrate mismatch and clinically significant glycemic excursions (19). Rigorous validation against reference values therefore remains essential before broader clinical implementation, particularly in culturally specific meal contexts that are underrepresented in the current literature.

Locally, a study by Chandran et al. (24) reported that 24.4% of adults with type 1 diabetes (n = 176) identified carbohydrate estimation as the most challenging aspect of diabetes self-management. To address this unmet need, NutriDish (Buzud Pte. Ltd., Singapore; iOS) (25) was developed as an AI-powered system for automated carbohydrate estimation from food images, designed with consideration for the diversity of Asian cuisines. However, its performance has not yet been formally evaluated against established reference standards, and no comparative data exists for newer generative AI models in this context.

This study therefore aimed to evaluate the accuracy of carbohydrate estimation for Asian cuisines using NutriDish and ChatGPT-5 (OpenAI, San Francisco, CA, USA) (26), benchmarked against dietitian-derived reference values. In addition, we assessed whether estimation accuracy met clinically acceptable thresholds of ± 10 g relative to reference values, providing a clinically meaningful framework for evaluating the practical utility of these tools in supporting diabetes self-management in a real-world Asian food environment.

## 2 Methods

### 2.1 Study design

This comparative study evaluated the accuracy of carbohydrate estimation using two AI-based tools, NutriDish and ChatGPT-5, benchmarked against dietitian-derived reference values. NutriDish is an AI-powered, image-based carbohydrate estimation system developed as a clinical decision support tool to facilitate carbohydrate counting among individuals with diabetes managed on MDII.

### 2.2 Food item selection and data collection

Food items were collected between July and August 2025 from a range of real-world dining settings in Singapore, including hawker centers, food courts, restaurants, and home environments, to ensure representation of the region's multicultural food landscape. Packaged food items with visible nutritional labels were additionally sourced from supermarkets, convenience stores, and grocery shops to represent commonly available commercial products.

Food items were categorized into two groups: simple food items, defined as single identifiable food components such as fruits, eggs, or plain rice; and composite meals, comprising multiple mixed or combined food components. Soup-based meals, beverages, and dishes mixed with sauces were excluded owing to the inherent difficulty of accurately estimating their portion sizes through visual assessment alone.

Prior to data collection, all study team members underwent standardized training covering meal photography techniques, multi-angle image acquisition, reference object placement, food weighing procedures, and documentation protocols, to ensure consistency across data collectors. All food images were captured under standardized conditions using an Apple iPhone 13 (Apple Inc., Cupertino, CA, USA), and two digital weighing scales of the same brand and model were used consistently throughout the data collection period to ensure measurement reliability. The images were subsequently analyzed with NutriDish, ChatGPT and carbohydrate content referenced with a dietician derived value.

### 2.3 Carbohydrate estimation procedures

For each food item, three independent carbohydrate estimates were obtained: one generated by NutriDish, one by ChatGPT-5, and one dietitian-derived reference value. Total carbohydrate content in grams served as the primary outcome measure.

#### 2.3.1 NutriDish

NutriDish is a mobile application that uses a retrieval-augmented generation (RAG) framework to identify food items in meal photographs, match them with a structured food composition knowledge base, deterministically calculate nutritional values from the retrieved entries, and generate clinically interpretable reports. This module employs an open-weight vision-language model that has been fine-tuned for the specific task of identifying one or more food items in meal photographs. The model accepts either a single meal image or dual-view meal images and outputs a structured list of food items, including food-category labels, standardized names, principal visible ingredients, broad cooking methods, confidence scores, and preliminary estimates for each item, including weight, projected area, estimated volume, carbohydrate, protein, fat, and energy content.

Its knowledge base, the BUZUD Nutrition Database, contains more than 2 million food entries derived from the US Department of Agriculture FoodData Central database (27) and locally curated Singapore food-composition data from the Health Promotion Board's Singapore Food Insights Database (28). Textual descriptions, visual features, and nutritional information are encoded as high-dimensional embedding vectors, enabling efficient semantic retrieval and matching of uploaded meal images with relevant nutritional records.

Figure 1 illustrates the NutriDish system workflow. The process begins in the mobile application, where users photograph their meals according to a standardized image-capture protocol comprising four key components: 1. inclusion of a predefined reference object for scale calibration, such as a standardized plate, bento box, Styrofoam container (Supplementary material 1), Singapore 50-cent coin, or Singapore 1-dollar coin (Supplementary material 2); 2. dual-angle image capture, consisting of a 90° top-down view for assessing surface area and a 45°-55° oblique view, captured from a distance of approximately 40–50 cm, to provide information on food depth and height; 3. clear and non-overlapping placement of individual food components; and 4. adherence to standardized image-quality requirements, including adequate lighting, minimal shadowing, and centralized framing of the meal. A preview of each captured image is subsequently displayed, allowing users to confirm its suitability or retake the photograph before uploading it using the “Save Image” function.

**Figure 1:**
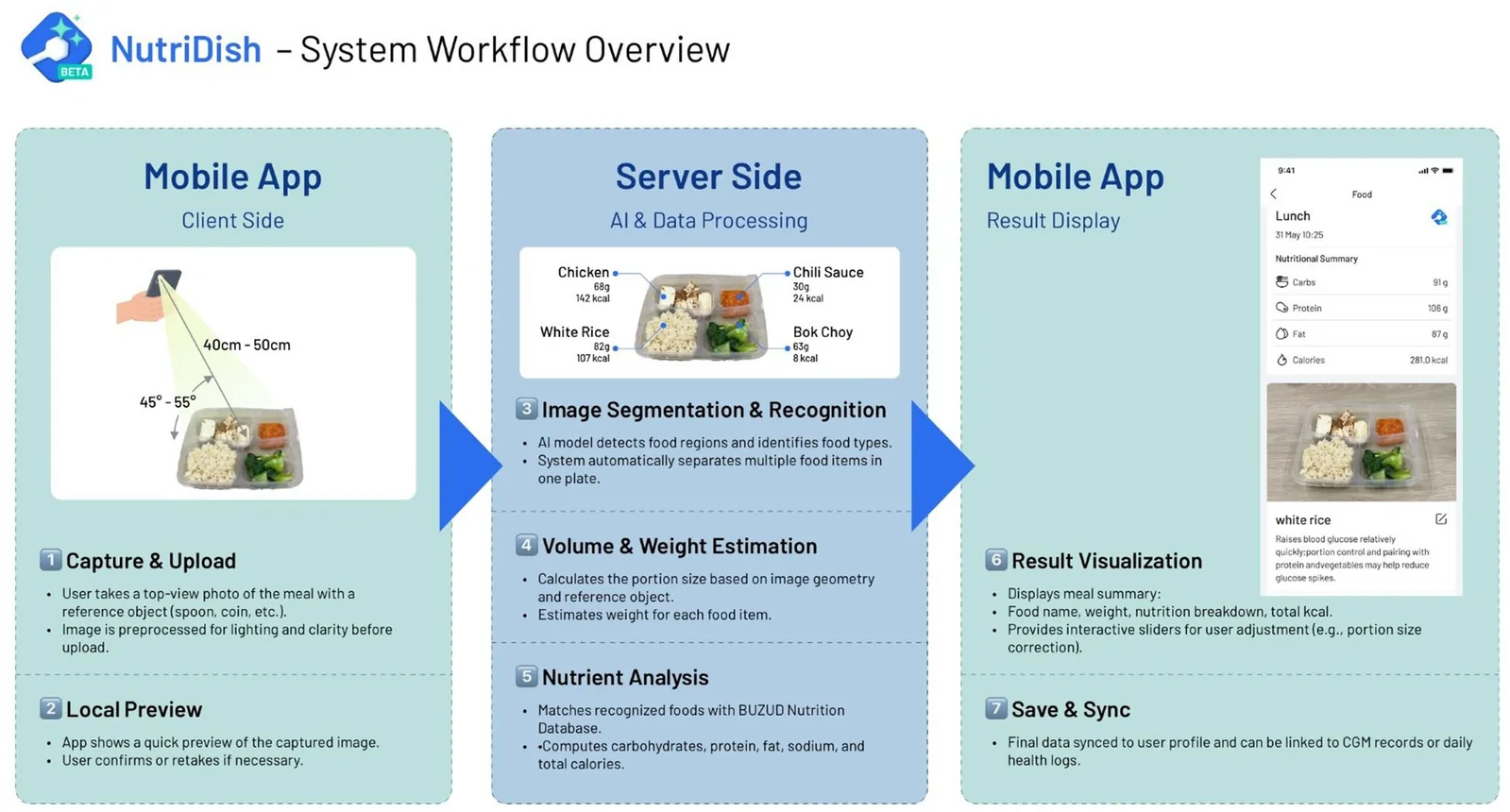
NutriDish—system workflow overview.

Following image upload, server-side computer vision algorithms detect and segment individual food components, including multiple items presented on the same plate. Visual characteristics, including shape, color, texture, and estimated portion size, are extracted from the top-down and oblique images. The top-down image provides information on surface area, whereas the oblique image contributes information on food depth and height. These complementary perspectives support volumetric estimation. The system then estimates portion volume and weight using image geometry calibrated against the predefined reference object.

The extracted multimodal feature embeddings are matched with entries in the BUZUD Nutrition Database through vector-similarity searching. The retrieved nutritional profiles are subsequently integrated with contextual information provided by the user, such as serving size, cooking method, and meal modifications. Based on these inputs, the system estimates energy, carbohydrate, protein, fat, sodium, and selected micronutrient content. A structured and clinically interpretable nutritional report is generated within approximately 30–50 sec.

The results are returned to the mobile application and presented as a meal summary containing the identified food items, estimated portion weights, nutrient composition, total carbohydrate content, total energy content, and personalized dietary recommendations. Interactive sliders allow users to review and correct the estimated portion sizes, with nutrient values recalculated accordingly. The resulting data can be synchronized with the user's profile and linked to continuous glucose monitoring records or daily health logs.

The algorithm was evaluated using approximately 5,000 food images representing a diverse range of Asian and Western meals, together with their corresponding reference nutritional information. This structured approach was designed to address an important limitation of image-only dietary assessment: the absence of scale references, standardized viewing angles, and contextual meal information may reduce the accuracy of portion-size estimation, particularly for mixed dishes and non-standard portions commonly encountered in Asian cuisines. Previous research evaluating generative AI for dietary assessment has similarly suggested that incorporating contextual meal information can improve carbohydrate-estimation accuracy compared with image-only approaches (20).

#### 2.3.2 ChatGPT-5

ChatGPT-5 is a multimodal LLM that functions as a Vision-Language Model capable of processing both text and image inputs (26). The system integrates computer vision and natural language processing to analyze food images, recognize food items, estimate portion sizes, and generate approximate nutritional estimates based on learned food representations and nutritional knowledge. Unlike rule-based carbohydrate calculators, ChatGPT-5 performs contextual image interpretation by analyzing visual features including color, texture, shape, food composition and relative portion dimensions.

Following image acquisition with NutriDish, the same meal image was uploaded to ChatGPT-5 between 8th August to 14th August 2025 via the ChatGPT web interface using a standardized prompt: “Please estimate the amount of carbohydrate.” The model was accessed between July and August 2025 via a ChatGPT Plus subscription, with the memory function enabled. A single response was recorded per image upload; repeated queries, prompt regeneration and iterative prompting were not performed. No additional system prompts, parameter modifications, or custom settings were applied beyond the default platform configuration. Carbohydrate estimates were generated within seconds of image upload.

#### 2.3.3 Dietitian-derived reference values

Dietitian-derived reference values served as the comparator standard for this study. Each food item, or each individual ingredient of composite meals, was weighed using calibrated digital kitchen scales accurate to the nearest gram. Reference carbohydrate values were calculated by a registered dietitian using measured food weights in conjunction with nutritional data from FoodWorks Online (29), the Health Promotion Board Singapore Energy and Nutrient Composition of Foods database (30), and the nBuddy—Nutritionist Buddy application (31). For packaged food items, carbohydrate content per 100 g was obtained directly from the product's nutritional label.

To minimize assessment bias, the dietitian responsible for calculating reference values was blinded to both NutriDish and ChatGPT-5 estimates throughout the process. An independent data recorder documented carbohydrate estimates from all three assessment methods in grams using a standardized data collection form, ensuring consistency in recording across all food items. The overall study procedure is illustrated in Figure 2.

**Figure 2:**
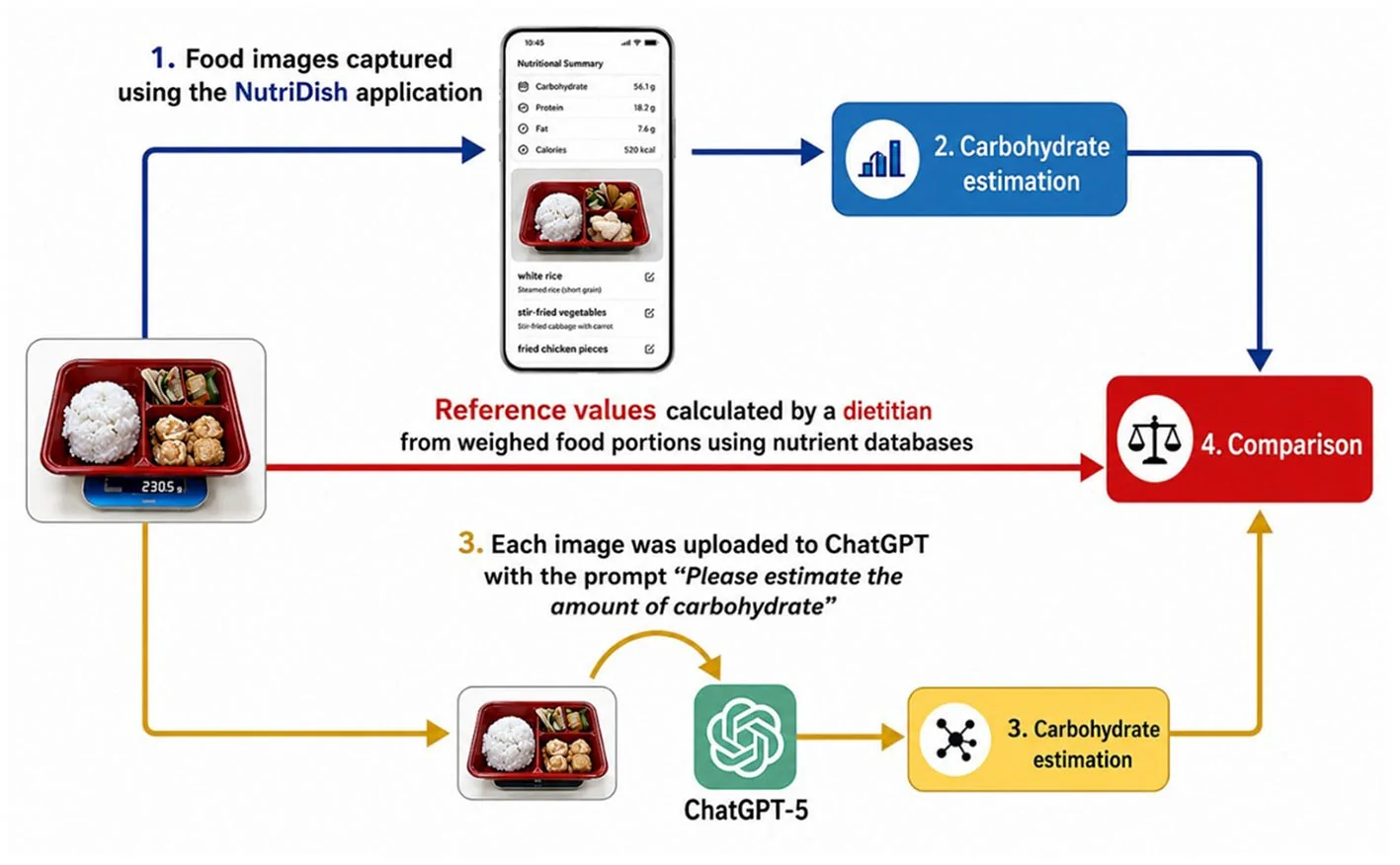
Outline of study procedure.

### 2.4 Study procedure

Figure 2 illustrates an outline of the study procedure. Food images were captured using the NutriDish application, with each meal photographed alongside a reference weight marker to generate a nutritional summary. Carbohydrate estimation was then performed using the application's built-in estimation function to produce app-derived values for each meal. Concurrently, each food image was uploaded to ChatGPT-5 with the standardized prompt and the model's estimates were recorded. Reference carbohydrate values were calculated independently by a qualified dietitian based on weighed food portions using established nutrient databases, serving as the gold standard for the study. The carbohydrate estimates derived from both the NutriDish application and ChatGPT-5 were subsequently compared against the dietitian-calculated reference values to evaluate the accuracy of each estimation method.

### 2.5 Ethical considerations

As the study did not involve human participants, patient recruitment, or collection of identifiable clinical data, formal ethical approval was not required.

### 2.6 Statistical analysis

The primary outcome was absolute error (AE), defined as the absolute difference between carbohydrate estimates generated by NutriDish or ChatGPT-5 and the dietitian-derived reference value for each food item. As the primary objective was to evaluate estimation accuracy relative to a reference standard rather than to formally establish superiority between methods, sample adequacy was assessed based on estimation precision. Variability estimates were informed by prior literature evaluating carbohydrate estimation accuracy, which reported a standard deviation of 16.5 g for the signed difference between estimated and reference carbohydrate values (19). Based on this estimate, a sample of 150 food items would yield a two-sided 95% confidence interval with a margin of error of approximately ± 2.6 g (total width ≈ 5.3 g), which was considered sufficient for evaluating the accuracy of carbohydrate estimation against the reference standard.

Distribution of carbohydrate estimates across the three assessment methods, NutriDish, ChatGPT-5, and dietitian-derived reference values, were summarized using appropriate descriptive statistics. Continuous variables were summarized using mean ± standard deviation (SD) and median with interquartile range (IQR). Categorical variables were summarized using frequency with group percentages. Accuracy metrics included absolute error (AE) and absolute percentage error (APE). These metrics were summarized using mean ± SD as the primary descriptive statistic, in keeping with convention in prior literature on automated carbohydrate estimation, with median (IQR) also reported for additional reference. The proportions within the clinically acceptable error thresholds of ± 10 g of the dietitian-derived reference values were compared using McNemar's test. Agreement between NutriDish or ChatGPT-5 and the dietitian-derived reference values was assessed using Bland-Altman analysis, with mean bias and 95% limits of agreement (LoA) reported. Proportional bias was further examined using linear regression of the difference against the mean of paired carbohydrate values.

Subgroup analyses were stratified by food complexity (simple food items vs. composite meals) and by the type of reference object used during estimation (plate, coin, nutritional label, or other) by NutriDish. Between-subgroup differences were assessed using the Wilcoxon rank-sum test (for two group comparisons) and Kruskal-Wallis test (for multiple group comparisons) for AE and APE, the chi-square or Fisher exact test for proportions within acceptable threshold, as appropriate. Where significant differences were identified, *post hoc* analyses were performed using Dunn's test for continuous variables and pairwise Fisher exact tests for categorical variables.

All statistical analyses were performed using R statistical software (version 4.5.1; R Core Team) (32). A two-sided *p*-value of less than 0.05 was considered statistically significant.

## 3 Results

### 3.1 Study characteristics

A total of 162 food items were collected between July and August 2025. Of these, 12 items were excluded as reference carbohydrate values could not be identified from the available nutritional databases, including certain cakes and buns (Supplementary material 4), resulting in 150 food items included in the final analysis.

Of the included food items, 90 (60.0%) were simple food items and 60 (40.0%) were composite meals. Reference objects used to facilitate carbohydrate estimation varied across food items. Standardized plates were the most frequently used reference object (*n* = 67, 44.7%), followed by nutritional labels (*n* = 44, 29.3%), coins (*n* = 21, 14.0%), and bento boxes or Styrofoam containers (*n* = 18, 12.0%).

### 3.2 Distribution of estimated carbohydrate values

Table 1 presents the distribution of carbohydrate estimations across the three methods. Both NutriDish and ChatGPT-5 produced slightly higher mean and median estimates than the reference across the overall sample of 150 food items. NutriDish estimated a mean of 36.0 ± 15.0 g and ChatGPT-5 38.0 ± 14.7 g, compared to the dietitian-derived reference of 35.2 ± 16.3 g. This pattern remained similar when stratified by dish complexity. For both simple and composite meal types, NutriDish and ChatGPT-5 estimated values were slightly higher than dietitian-derived values.

**Table 1:** Distribution of carbohydrate estimation (in grams) by NutriDish, ChatGPT-5, and dietitian-derived reference values.

Metric	NutriDish	ChatGPT-5	Reference values
Overall (*n* = 150)			
Mean ± SD	36 ± 15	38 ± 14.7	35.2 ± 16.3
Median (IQR)	34.0 (27.0–45.0)	36.0 (30.0–47.9)	32.9 (26.0–44.0)
Type of dish			
Simple foods items (*n* = 90)			
Mean ± SD	29.8 ± 11.4	32.9 ± 12.4	28.9 ± 11.7
Median (IQR)	30.0 (24.3–39.0)	31.8 (27.0–39.8)	29.9 (21.0–36.3)
Composite meals (*n* = 60)			
Mean ± SD	45.3 ± 15	45.6 ± 14.7	44.5 ± 17.8
Median (IQR)	45.7 (34.8–57.3)	47.3 (35.0–52.3)	40.5 (30.0–60.3)
Reference objects			
Plate (*n* = 67)			
Mean ± SD	37.9 ± 16.6	38.4 ± 15.8	37.2 ± 18.2
Median (IQR)	37.2 (27.0–50.0)	37.0 (27.3–50.0)	36.0 (24.0–50.0)
Coin (*n* = 21)			
Mean ± SD	36.9 ± 20.3	37.9 ± 20.4	34.3 ± 21.2
Median (IQR)	35.0 (20.0–45.0)	33.0 (20.0–47.5)	32.0 (14.0–44.0)
Nutritional label (*n* = 44)			
Mean ± SD	33.1 ± 8.4	36 ± 7.2	31.6 ± 7.8
Median (IQR)	30.3 (29.4–40.0)	36.0 (30.0–40.4)	30.0 (28.7–36.0)
Others (bento boxes and styrofoam containers) (*n* = 18)			
Mean ± SD	34.7 ± 13.8	41.5 ± 16.5	37.4 ± 17.8
Median (IQR)	34.5 (23.3–42.4)	40.7 (31.0–51.8)	35.0 (22.5–45.0)

SD, standard deviation; IQR, interquartile range; n, number of observations.

When stratified by reference object, both methods also produced slightly higher estimates than the reference for items photographed with a plate, coin, and nutritional label. The exception was the bento box and Styrofoam container subgroup, where NutriDish estimated values were slightly lower than the reference while ChatGPT-5 estimated values were higher.

### 3.3 Comparative accuracy of carbohydrate estimation between NutriDish and ChatGPT-5 against dietitian-derived reference values

Table 2 presents the accuracy and agreement metrics for NutriDish and ChatGPT-5 against the dietitian-derived reference values. Given the non-normal distribution of both metrics, which are bounded at zero and right-skewed by nature, confirmed using the Shapiro-Wilk test, comparisons were made using the Wilcoxon signed-rank test. The mean absolute error (MAE) was significantly lower for NutriDish than for ChatGPT-5 [5.9 ± 5.3 g (median: 4.1 g, IQR: 1.0–9.0) vs. 8.7 ± 7.4 g (median: 6.0 g, IQR: 3.0–13.4); *p* < 0.001]. Consistent with these findings, the mean absolute percentage error (MAPE) was also significantly lower for NutriDish than for ChatGPT-5 [22.1 ± 35.7% (median: 13.0%, IQR: 4.9–25.0) vs. 32.9 ± 50.8% (median: 22.1%, IQR: 9.1–37.1); *p* < 0.001].

**Table 2:** Carbohydrate estimation accuracy of NutriDish and ChatGPT-5, and effect of food complexity on NutriDish performance.

Metric	Absolute error (AE)		Absolute percentage error (APE)		Proportion within +/-10g
	Mean ±SD (g)	Median (IQR) (g)	Mean ±SD (%)	Median (IQR) (%)	Within +/-10 g, *n* (%)
NutriDish	5.9 ± 5.3	4.1 (1.0–9.0)	22.1 ± 35.7	13.0 (4.9–25.0)	121 (80.7%)
ChatGPT5	8.7 ± 7.4	6.0 (3.0–13.4)	32.9 ± 50.8	22.1 (9.1–37.1)	104 (69.3%)
*p* value		< 0.001		< 0.001	0.017
Simple food items (*n* = 90)	4.1 ± 4.3	3 (0.3 to 7)	21 ± 39.1	11.4 (1 to 23.3)	82 (91.1%)
Composite meals (*n* = 60)	8.6 ± 5.6	8.8 (4 to 12)	23.8 ± 30.1	17 (9.9 to 25.9)	39 (65%)
*p* value		< 0.001		0.008	< 0.001

Statistical comparisons between NutriDish and ChatGPT-5 were performed using the Wilcoxon signed-rank test for AE and APE, and McNemar's test for proportion within ± 10 g. Statistical comparisons between simple and composite food items were performed using the Wilcoxon rank-sum test for AE and APE and chi-square test for proportion within ± 10 g. Non-parametric tests were used given the non-Normal distribution of AE and APE, and both mean with SD and median with IQR were presented as summary statistics to provide the comprehensive description. AE, Absolute Error; APE, Absolute Percentage Error; SD, Standard Deviation; IQR, Interquartile Range.

A significantly greater proportion of NutriDish estimates fell within clinically acceptable error thresholds of ± 10 g of the dietitian-derived reference values compared to ChatGPT-5: 80.7% versus 69.3% (*p* = 0.017). Of the 150 food items, 90 (60.0%) were classified as acceptable for both methods, while 15 (10.0%) were unacceptable for both (i.e., outside ± 10 g threshold). NutriDish achieved acceptable estimates in 31 (20.7%) cases where ChatGPT-5 did not, compared with 14 (9.3%) cases where ChatGPT-5 achieved acceptable estimates but NutriDish did not.

Bland-Altman analysis further confirmed the closer agreement of NutriDish with the dietitian-derived reference values (Figure 3). NutriDish demonstrated a small, non-significant mean bias of 0.80 g (95% CI: −0.49 to 2.08; *p* = 0.222), suggesting no evidence of systematic bias at the group level (Figure 3A). In contrast, ChatGPT-5 exhibited a statistically significant mean bias of 2.79 g (95% CI: 1.01 to 4.57; *p* = 0.002), suggesting a tendency toward systematic overestimation of carbohydrate content relative to the reference standard (Figure 3B). At the individual level, NutriDish demonstrated narrower limits of agreement compared to ChatGPT-5, indicating greater consistency in individual-level estimates.

**Figure 3:**
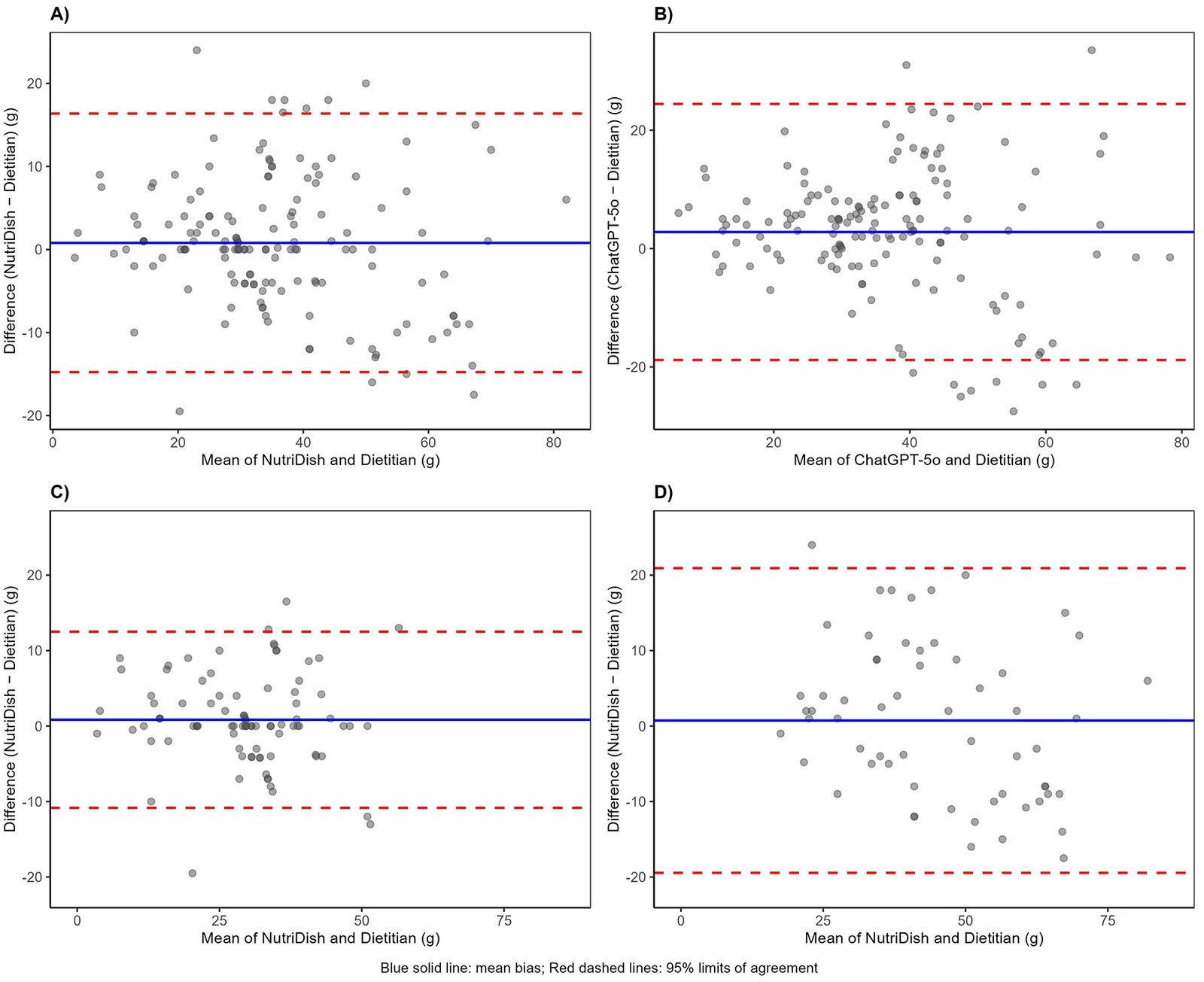
Bland-Altman plots: automated methods vs. dietitian-derived reference. **(A)** NutriDish vs. Dietitian (overall). **(B)** ChatGPT-50 vs. dietitian (overall). **(C)** NutriDish vs. dietitian (simple items). **(D)** NutriDish vs. dietitian (composite dishes).

Assessment of proportional bias revealed a small but statistically significant negative slope for NutriDish (slope = −0.09, 95% CI: −0.175 to −0.007; *p* = 0.034), suggesting a tendency toward greater underestimation at higher carbohydrate values. For ChatGPT-5, the evidence for proportional bias was weaker and did not reach statistical significance (slope = −0.12, 95% CI: −0.241 to 0.003; *p* = 0.055). However, the slopes for both methods were small in magnitude, and the observed proportional bias is unlikely to be of clinical significance across the carbohydrate range encountered in this study.

Given NutriDish's superior overall performance relative to ChatGPT-5, further subgroup analyses were conducted stratified by dish complexity and reference object type.

### 3.4 Subgroup analyses: accuracy and agreement of NutriDish carbohydrate estimation by dish complexity

Table 2 also presents the accuracy and agreement metrics for NutriDish stratified by dish complexity. NutriDish demonstrated significantly greater accuracy in estimating carbohydrates for simple food items than for composite meals across all metrics. For simple dishes (*n* = 90), the MAE was significantly lower (4.1 ± 4.3 g; median: 3.0 g; IQR: 0.3–7.0) compared to composite meals (8.6 ± 5.6; median: 8.8 g; IQR: 4.0–12.0; *p* < 0.001). Similarly, the MAPE was significantly lower for simple items compared to composite dishes [21.0 ± 39.1% vs. 23.8 ± 30.1%; median: 11.4% (IQR: 1.0–23.3) vs. 17.0% (IQR: 9.9–25.9); p = 0.008]. The proportion of estimates falling within clinically acceptable error thresholds was consistently higher for simple food items than for composite meals: 91.1% vs. 65.0% within ± 10 g (*p* < 0.001). Collectively, these findings indicate that dish complexity substantially affects the accuracy of AI-based carbohydrate estimation, with composite meals posing greater challenges for component identification and portion-size recognition.

Bland-Altman analysis showed comparable and non-significant mean bias for both simple items (Figure 3C) and composite meals (Figure 3D) [0.84 g (95% CI: −0.41 to 2.08; *p* = 0.186) and 0.73 g (95% CI: −1.93 to 3.40; *p* = 0.583), respectively]. However, the limits of agreement were notably narrower for simple items compared to composite dishes, indicating considerably greater variability in individual-level estimates for composite meals.

Collectively, these findings suggest that while NutriDish demonstrated unbiased estimation across both dish types, its accuracy and consistency were substantially better for simple dishes compared to composite dishes, which is consistent with the inherently greater difficulty in estimating carbohydrate content of multi-component complex dishes.

### 3.5 Subgroup analysis: accuracy and agreement of NutriDish carbohydrate estimation by reference object type

The accuracy of NutriDish carbohydrate estimation differed significantly across reference object types (Table 3). MAE varied significantly by reference object (*p* < 0.001), with nutritional label images yielding the lowest MAE at 2.7 ± 4.2 g (median: 0.1 g, IQR: 0.0–4.1) and coin images yielding the highest at 9.3 ± 7.3 g (median: 8.0 g, IQR: 3.0–15.0). Images using a plate, bento box, or Styrofoam container as the reference object demonstrated intermediate levels of accuracy, with MAEs ranging from 6.7 ± 4.4 g to 6.9 ± 4.8 g.

**Table 3:** Comparison of NutriDish carbohydrate estimation errors against reference values across reference object types.

Metric	Plate (*n* = 67)	Coin (*n* = 21)	Nutritional label (*n* = 44)	Others (*n* = 18)	*p* value
Absolute error (AE)					
Mean ± SD (g)	6.7 ± 4.4	9.3 ± 7.3	2.7 ± 4.2	6.9 ± 4.8	
Median (IQR) (g)	7.0 (3.6 to 9.0)^a^	8.0 (3.0 to 15.0)^a^	0.1 (0 to 4.1)^b^	4.9 (3.0 to 9.8)^a^	< 0.001
Absolute percentage error (APE)					
Mean ± SD (%)	23.4 ± 25.8	48.1 ± 74.6	9.0 ± 14.5	19.1 ± 14.7	
Median (IQR) (%)	16.7 (10.6 to 26.5)^a^	21.1 (9.1 to 53.1)^a^	0.3 (0 to 12.3)^b^	14.5 (9.9 to 22.8)^a^	< 0.001
Within ± 10 g, *n* (%)	55 (82.1%)	12 (57.1%)	40 (90.9%)	14 (77.8%)	0.017

Overall *p*-values from Kruskal-Wallis test for AE and APE, and Fisher's exact test for proportion within ± 10 g. ^a, b^Groups sharing the same superscript letter do not differ significantly on *post-hoc* Dunn's test with Bonferroni correction (*p* > 0.05). Nutritional Label (b) differs significantly from Plate, Coin, and Others (a) for both absolute error and absolute percentage error (all *p* ≤ 0.002). Both mean with SD and median with IQR of error metrics were presented to provide a comprehensive description of the data.

A consistent pattern was observed for MAPE, which ranged from 9.0 ± 14.5% for nutritional label images (median: 0.3%, IQR: 0.0–12.3) to 48.1 ± 74.6% for coin images (median: 21.1%, IQR: 9.1–53.1) (*p* < 0.001). The proportion of estimates meeting clinically acceptable error thresholds also differed significantly across reference object types at ± 10 g (*p* = 0.017). Nutritional label images achieved the highest proportions within these thresholds (90.9%), while coin images showed the lowest (57.1%). Collectively, these findings suggest that the choice of reference object substantially influences the accuracy of AI-based carbohydrate estimation, with nutritional labels providing the most reliable contextual information for portion-size recognition and coin images the least reliable.

Bland-Altman analysis further supported these findings (Figure 4). Plate images showed a mean bias of 0.7 g (95% CI −1.26 to 2.66, LoA −15.04 to 16.43 g, total spread 31.47 g; Figure 4A), while coin images demonstrated the widest limits of agreement (LoA −20.23 to 25.52 g, total spread 45.75 g; Figure 4B). The narrowest limits of agreement were observed for nutritional label images (LoA −7.95 to 10.97 g, total spread 18.91 g; Figure 4C), where a statistically significant bias was also observed (bias 1.51 g, 95% CI 0.04–2.98, *p* = 0.044). For others (bento box and Styrofoam container images), the mean bias was −2.74 g (95% CI −6.77 to 1.28, LoA −18.60 to 13.11 g, total spread 31.71 g; Figure 4D). Biases for plate, coin, and bento box/Styrofoam container subgroups did not differ significantly from zero.

**Figure 4:**
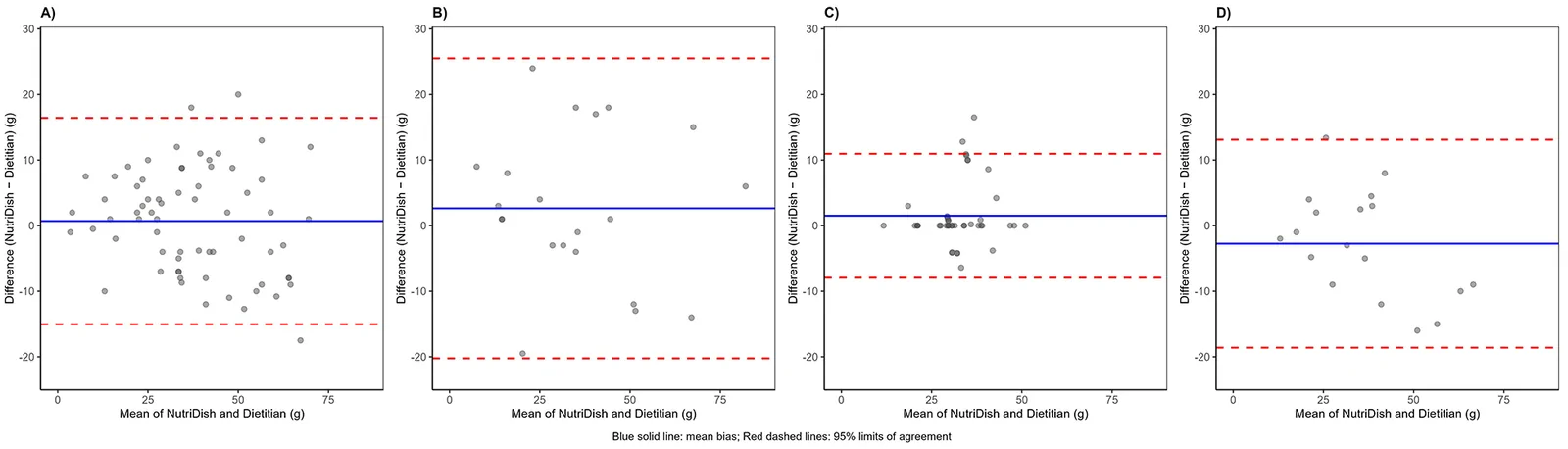
Bland-Altman plots: NutriDish vs. dietitian, by reference object. **(A)** Plate, **(B)** Coin, **(C)** Nutritional Label, **(D)** Others.

## 4 Discussion

### 4.1 NutriDish as a clinically viable carbohydrate estimation tool

This study evaluated the performance of NutriDish and ChatGPT-5 for image-based carbohydrate estimation across 150 food items representing diverse ethnic cuisines. NutriDish achieved substantially lower errors than ChatGPT-5 across all accuracy metrics, suggesting that a purpose-built carbohydrate estimation system may perform more reliably than a general-purpose LLM for nutritional assessment tasks.

These results are consistent with emerging evidence in the field. Johansen et al. (20) reported that ChatGPT-4o demonstrated systematic bias for composite meals and foods without a size reference, exhibiting both overestimation and underestimation of carbohydrate content. Similarly, Tecce et al. (23) evaluated ChatGPT-4o and Gemini Advanced across varying levels of contextual meal information and found that estimation accuracy improved substantially when ingredient lists and food details were provided; ChatGPT-4o achieved a MAPE of 18.1% under moderate contextual conditions and 13.7% when full ingredient details were available. Nicola et al. similarly stated that both ChatGPT and Gemini improved their estimation accuracy when provided with more contextual data. In comparison, NutriDish achieved a MAE of 5.9 g and MAPE of 22.1%, suggesting that a diabetes-specific architecture incorporating standardized image capture protocols, contextual reference objects, and structured food recognition may provide more consistent carbohydrate estimation performance in real-world settings. Taken together, these findings indicate that domain-specific design incorporating structured nutritional databases, portion-size modeling and image-calibration features confers meaningful advantages over general-purpose AI systems for clinical nutritional assessment.

### 4.2 The role of structured image acquisition

The structured meal capture protocol used in NutriDish likely contributed substantially to the observed improvement in estimation accuracy. Unlike unstructured image acquisition approaches commonly used in general-purpose multimodal AI systems, NutriDish standardized meal image capture through the use of predefined reference objects, dual-angle photography (45° and 90°), and clear positioning of food items without overlap. These measures likely reduced image variability, improved food segmentation, and enhanced depth perception and volume approximation compared with single-view image analysis. Standardized scale calibration may further reduce errors related to hidden ingredients and complex mixed meals, thereby improving estimation consistency and reproducibility across diverse meal types.

These methodological distinctions are particularly relevant when contextualizing the performance gap between NutriDish and general-purpose multimodal AI systems. Johansen et al. (20) reported substantially reduced accuracy for composite meals using ChatGPT-4o, with MAE ranging from 13.9 g to 17.5 g and only 43.3%-46.7% of estimates falling within ± 10 g of reference values. The authors attributed these limitations to single-view imaging, lack of depth estimation, overlapping meal components, and the absence of structured meal capture protocols—all of which NutriDish was specifically designed to address. Furthermore, NutriDish was optimized for mixed Asian meals, which may additionally explain its superior performance in composite meal analysis.

### 4.3 Estimation accuracy across food complexity and reference objects

Dish complexity significantly influenced estimation accuracy. NutriDish demonstrated lower MAE for simple food items than for composite meals, indicating that meals containing multiple ingredients, variable preparation methods, or concealed components remain challenging for image-based estimation systems. These findings are consistent with limitations reported in evaluations of GoCARB (17) and other image-based carbohydrate estimation tools (22). They are particularly relevant in Asian food environments, where composite meals with hidden ingredients, sauces, and mixed preparation styles are prevalent and may introduce greater uncertainty in estimation. Prior work has further demonstrated that estimation errors are more frequent and pronounced for meals containing very high or very low amounts of carbohydrates (7), and that carbohydrate content in large meals tends to be underestimated while that in small meals tends to be overestimated (11)—patterns that were similarly observed in this study.

Reference object selection also substantially affected performance. Nutritional labels produced the lowest MAE; however, the carbohydrate values on the labels were not visible during image capture. The improved accuracy observed for packaged foods is therefore unlikely to reflect direct label reading and may instead suggest that NutriDish has been trained on a dataset that includes commonly available packaged food products, enabling more reliable recognition and estimation for these items. This distinction has implications for generalizability, as performance for packaged foods may reflect familiarity within the training dataset rather than a transferable advantage applicable to all labeled products. In contrast, coins demonstrated the highest MAE, possibly owing to their limited spatial context and susceptibility to photographic distortion. Taken together, these findings suggest that estimation accuracy depends not only on the underlying algorithm, but also on the quality and standardization of image acquisition.

### 4.4 Systematic bias and its implications for insulin dosing

A notable finding was the systematic underestimation of carbohydrate content at higher meal carbohydrate levels and overestimation at lower levels. This directional bias carries meaningful clinical implications: underestimation of carbohydrate-dense meals may lead to insulin underdosing and suboptimal postprandial glycemic control, while overestimation of smaller meals may increase hypoglycemia risk (13, 33, 34). Although overall MAE remained low, aggregate accuracy metrics alone may obscure these clinically relevant patterns.

These concerns are compounded by the multifactorial nature of postprandial glucose excursions. Parillo et al. (35) demonstrated that postprandial glucose excursion was higher with high glycemic index meals even when an accurate insulin-to-carbohydrate ratio was applied. Additionally, Cobry et al. (36) showed that a lower postprandial glucose excursion was experienced when insulin injection 20 min before meals reduced postprandial excursions compared to peri-or postprandial injections. Beyond carbohydratre quantity, metabolic responses are also influenced by endogenous insulin secretion, insulin resistance, dietary protein and fat content (37, 38). These factors are particularly relevant in obesity, prediabetes, and type 2 diabetes, where compensatory hyperinsulinemia may further alter postprandial responses. Therefore, AI-based carbohydrate estimation should complement, rather than replace, broader metabolic assessment incorporating meal composition, glycemic responses, and individual metabolic characteristics.

## 5 Study limitations

Several limitations of this study warrant acknowledgment.

First, the relatively modest sample size and controlled image-capture conditions may not fully reflect real-world variability in ambient lighting, camera angles and meal presentation styles, potentially limiting the ecological validity of the findings. Future studies should incorporate a broader range of real-world imaging conditions to better assess performance under everyday use.

Second, the study evaluated estimation accuracy as the primary outcome without assessing downstream clinical effects, such as insulin dosing decisions or postprandial glycemic control. The system's exclusive focus on carbohydrate estimation is also a notable constraint, as protein and fat independently influence postprandial glucose excursions (37, 38). In addition, endogenous insulin secretion also influences postprandial glucose levels. Jones et al. (39) demonstrated that patients with lower endogenous insulin secretion experienced greater postprandial hyperglycaemia and showed a greater glycaemic response to prandial exogenous insulin. The incorporation of full macronutrient composition may be particularly important for patients using hybrid closed-loop insulin delivery systems, where accurate meal composition data are essential for optimizing automated insulin delivery algorithms. Thus, future studies should not only include these factors into glucose estimation but also examine the impact of these factors on insulin dosing and postprandial glucose levels.

Third, the reference standard employed, namely weighed food portions combined with dietitian-administered nutrient database estimates, while widely accepted in nutritional research, does not constitute a true biochemical gold standard. This limitation is especially relevant for mixed dishes and local hawker foods such as economy rice (a customizable mixed-rice dish where diners select from an array of pre-cooked dishes served over steamed rice), laksa, and nasi padang, where variability in ingredients and cooking methods may introduce systematic error into reference values. For certain food items, reference values could not be assigned due to the absence of corresponding entries in validated nutrient databases. Future studies should consider direct chemical analysis or triangulation with multiple validated dietary assessment methods to strengthen reference accuracy.

Fourth, a direct head-to-head comparison with other carbohydrate estimation tools was not feasible as GoCARB and SNAQ were unavailable for download in our region during the study period. Furthermore, existing systems have mainly been evaluated using structured European or Western meals. Their performance may not generalize to multi-ethnic Asian cuisines, which frequently includes composite dishes, overlapping ingredients, sauces and gravies. NutriDish was specifically developed for Chinese, Malay, Indian, and mixed Singaporean meals using locally relevant food-composition data and user-editable food and portion estimates. Cross-study numerical comparisons should be interpreted cautiously because of differences in cuisines, datasets, reference standards, image-acquisition protocols, and outcome measures. Future studies should directly compare NutriDish with accessible specialized applications using the same Asian meal dataset and dietitian-derived reference standard.

Fifth, the study did not explore alternative prompting strategies for ChatGPT-5 as a single standardized prompt was used to ensure consistency and comparability across all meal images. However, this approach represents only one prompting strategy and may not reflect ChatGPT's best possible performance. Alternative strategies, such as providing cuisine-specific context, reference objects, portion-size information, few-shot examples, stepwise instructions, or opportunities for follow-up clarification, could potentially improve food recognition and carbohydrate estimation. Conversely, overly detailed or leading prompts might introduce bias or reduce consistency. As prior studies have demonstrated, prompt optimization can meaningfully improve AI performance on nutrition estimation tasks (23), and future work should systematically evaluate the effects of varied prompting approaches.

Finally, as the dataset comprised Singapore local cuisine exclusively, these findings may not be as generalizable to other food cultures or geographic settings. While the dataset encompassed diverse ethnic cuisines reflecting Singapore's multicultural culinary landscape, this food environment, characterized by hawker center dining, communal eating and dishes not well represented in international food databases, presents unique challenges for external validation. Utilizing more diverse and internationally recognized datasets, such as Nutrition5K, could improve model inclusivity and generalizability. Independent external validation across different geographic and cultural contexts, alongside broader community-based validation within Singapore, will be necessary before wider clinical deployment can be recommended.

## 6 Clinical implications and future directions

These findings demonstrate that NutriDish is a clinically viable adjunct for carbohydrate estimation in diabetes self-management. Its relatively low estimation error may reduce the cognitive burden of carbohydrate counting and support more consistent prandial insulin-dose calculations. However, it should remain a decision-support tool requiring user verification and clinical oversight, particularly for complex meals and higher carbohydrate loads. Its ability to estimate protein, fat, fiber, and energy intake may also support weight management and nutritional screening.

In NutriDish, the estimated carbohydrate content automatically populates and syncs directly with the personalized insulin-dose calculator. Therefore, a lower absolute estimation error enhances the precision of prandial insulin dosing. For example, for an insulin-to-carbohydrate ratio of 1 unit per 10 g, a 10-g carbohydrate-estimation error would translate into an approximately 1 unit of insulin-dose error. Precise insulin dosing mitigates over-titration, which can trigger postprandial hypoglycaemia, and under-titration, which leads to postprandial hyperglycaemia. Over time, this optimization may improve Time-in-Range (TIR) and overall glycaemic control. However, the present study assessed carbohydrate-estimation accuracy rather than insulin-dosing or clinical outcomes. Consequently, these benefits remain potential implications requiring validation in prospective, real-world studies.

Future research should conduct head-to-head studies comparing NutriDish estimates with actual carbohydrate values determined through laboratory-based macronutrient analysis, which would provide a more objective reference standard than dietitian-derived estimates. Studies should also evaluate clinically significant errors and their effects on insulin-dosing safety, postprandial glycaemia, and patient-reported outcomes in real-world settings. Future development should improve composite-meal and portion-size recognition and incorporate protein, fat, fiber, meal composition, endogenous insulin secretion, insulin dynamics, continuous glucose-monitoring trends and active insulin information to further individualize insulin-dose recommendations.

## 7 Conclusion

NutriDish achieved a lower MAE and more consistent agreement with dietitian-derived reference values than ChatGPT-5 across diverse ethnic meals. Its structured image-acquisition workflow, incorporation of dual-angle photography, predefined reference objects, and standardized food positioning, likely contributed to its superior performance. Nevertheless, reduced accuracy for composite meals, coin-based calibration, and higher-carbohydrate meals highlights the need to interpret overall accuracy within a clinically meaningful context.

These findings demonstrate that NutriDish is a clinically viable digital adjunct for carbohydrate estimation and prandial insulin dose adjustment in diabetes self-management. Nonetheless, user verification and professional clinical oversight remain imperative, particularly when evaluating complex composite meals or high carbohydrate loads. Future longitudinal studies should evaluate its direct effects on insulin dosing safety and glycemic outcomes, incorporate risk-based analyses of estimation errors, and externally validate the system across diverse populations and broader food environments.

## Data Availability

The original contributions presented in the study are included in the article/Supplementary material, further inquiries can be directed to the corresponding author.
