# Supplementary material for: Comparative accuracy of automated carbohydrate estimation using NutriDish and ChatGPT-5 against dietitian-derived reference values
Source: Front Nutr. 2026 Sep 29;13:1927840. doi: 10.3389/fnut.2026.1927840 (PMC13660361; doi:10.3389/fnut.2026.1927840)
Supplement: Supplementary file 1 [file Table_1.docx]

**Supplementary Material 1**

The NutriDish system supports built-in reference objects including personalized plates, bento boxes, and Styrofoam containers commonly used in food courts, canteens, and hawker centers.
