# Supplementary material for: Comparative accuracy of automated carbohydrate estimation using NutriDish and ChatGPT-5 against dietitian-derived reference values
Source: Front Nutr. 2026 Sep 29;13:1927840. doi: 10.3389/fnut.2026.1927840 (PMC13660361; doi:10.3389/fnut.2026.1927840)
Supplement: Supplementary file 2 [file Table_2.docx]

**Supplementary Material 2**

*This material contains screengrabs from the NutriDish mobile application. The NutriDish module was co-designed by the research team in collaboration with an industry partner under a formal research collaboration agreement.*

When the built-in reference objects such as personalized plates, bento boxes, and Styrofoam containers commonly used in food courts, canteens, and hawker centers these were unavailable, a Singapore 50-cent or 1-dollar coin was placed beside the meal as an alternative reference object.
