# Supplementary material for: Comparative accuracy of automated carbohydrate estimation using NutriDish and ChatGPT-5 against dietitian-derived reference values
Source: Front Nutr. 2026 Sep 29;13:1927840. doi: 10.3389/fnut.2026.1927840 (PMC13660361; doi:10.3389/fnut.2026.1927840)
Supplement: Supplementary file 3 [file Table_3.docx]

**Supplementary Material 3**

*This material contains screengrabs from the NutriDish mobile application. The NutriDish module was co-designed by the research team in collaboration with an industry partner under a formal research collaboration agreement.*

Image capture proceeded only when the application's frame indicators confirmed acceptable image quality by turning green.
