# Supplementary material for: Comparative accuracy of automated carbohydrate estimation using NutriDish and ChatGPT-5 against dietitian-derived reference values
Source: Front Nutr. 2026 Sep 29;13:1927840. doi: 10.3389/fnut.2026.1927840 (PMC13660361; doi:10.3389/fnut.2026.1927840)
Supplement: Supplementary file 4 [file Table_4.docx]

**Supplementary Material 4**

The following 12 food items were excluded as reference carbohydrate values could not be identified from the available nutritional databases, including certain cakes and buns.

(1) Chocolate Cake: Excluded for analysis because Ingredient composition could not be determined; therefore, corresponding nutritional information could not be retrieved from the nutritional database.

(2) Earl Grey Cake: Excluded for analysis because the ingredient composition could not be determined; therefore, corresponding nutritional information could not be retrieved from the nutritional database.

(3) Early Grey Scone: Excluded for analysis because Ingredient composition could not be determined; therefore, corresponding nutritional information could not be retrieved from the nutritional database.

(4) Kimchi Pancake: Excluded for analysis because Ingredient composition could not be determined; therefore, corresponding nutritional information could not be retrieved from the nutritional database.

(5) Fan Choy: Excluded from analysis because the sauce was mixed with the rice, preventing accurate quantification of individual components.

(6) Tortilla pizza: Excluded for analysis because Ingredient composition could not be determined; therefore, corresponding nutritional information could not be retrieved from the nutritional database.

(7) Lontong: Excluded from analysis because the gravy obscured the food components, and substantial overlap among food items prevented adequate visualization and identification of individual ingredients.

(8) Lontong: Excluded from analysis because the gravy and sauce obscured the food components, and substantial overlap among food items prevented adequate visualization and identification of individual ingredients.

(9) Hor Fun: Excluded due to overlapping food items, which hindered accurate assessment.

(10) Yong Tau Foo: Excluded from analysis because overlapping food items and ingredients submerged in the soup could not be clearly visualized, preventing accurate assessment.

(11) Mee Bandung: Excluded due to overlapping food items and an inability to accurately estimate the quantity of sauce.

(12) Spaghetti: Excluded due to overlapping food items and an inability to accurately estimate the quantity of sauce.

Fried Carrot Cake #210

Macaroni #187
